# The evolution of object detection from CNNs to transformers and multi-modal fusion

**DOI:** 10.1038/s41598-026-37052-6

**Published:** 2026-02-05

**Authors:** Zeran Wang, Yuan Chen, Yuhao Gu, Jian Liu, Xudong Zhu, Mianwang He

**Affiliations:** 1 School of Computer and Communication Engineering, University of Science and Technology Beijing, Beijing, 100083 China; 2 University of Bristol, Bristol, BS8 1QU UK; 3 School of Economics and Management, Beijing University of Posts and Telecommunications‌, Beijing, 100876 China; 4 People’s Liberation Army General Hospital, Beijing, 100039 China

**Keywords:** Object detection, CNN, Transformer, Multi-modal feature fusion, Engineering, Mathematics and computing

## Abstract

Object detection, a cornerstone of computer vision, aims to localize and classify objects within images. This comprehensive survey reviews modern object detection methods, focusing on two dominant paradigms: Convolutional Neural Networks (CNNs) and Transformer-based architectures. This work provides a structured comparison of CNN-based and Transformer-based detection paradigms, highlighting their complementary strengths and trade-offs. CNNs demonstrate advantages in local feature extraction and computational efficiency, whereas Transformers excel at capturing global context through self-attention mechanisms. We also analyze multi-modal fusion techniques integrating Red-Green-Blue (RGB), Light Detection and Ranging (LiDAR), and language embeddings. Benchmark results from representative models include: Real-Time Detection Transformer (RT-DETR) achieves 53.1% mean Average Precision (mAP) at Intersection over Union (IoU) at 0.5 : 0.95, You Only Look Once version 8 (YOLOv8) achieves 50.2% mAP at 0.5:0.95, real-time detectors exceed 100 frames per second (FPS) with competitive accuracy, and specialized infrared methods achieve 92.45% F-measure on NUAA-SIRST dataset. The work introduces a novel taxonomy of multi-modal fusion strategies, documents field-wide and review-specific limitations, and synthesizes recent 2024 to 2025 benchmarks across diverse datasets. Despite these advances, significant challenges remain in handling scale variation, occlusion effects, and domain adaptation. This survey outlines these persistent obstacles and promising research directions, providing a structured reference for researchers and practitioners.

## Introduction

Object detection, a fundamental computer vision task, aims to identify and localize objects of interest within digital images or video streams. This technology supports a wide array of real-world applications across multiple domains. In autonomous driving, state-of-the-art methods such as TransFuser employ multi-modal fusion transformers to integrate image and LiDAR representations through cross-attention mechanisms^1^. In surveillance systems, multiple specialized approaches address diverse detection scenarios: enhanced learning frameworks enable moving object detection in challenging video scenes, thermal video surveillance methods leverage feature pooling modules for robust analysis, and multi-scale contrast preserving encoder-decoder architectures support local change detection from thermal video^2^. In medical imaging, specialized deep learning models such as Multi-Organ and Lesion Segmentation Network (MOLS-Net) enable automated segmentation and detection of critical anatomical abnormalities, including aortic dissection identification from computed tomography (CT) volumes, through lightweight frameworks that combine efficient feature extraction and fusion^3^. In robotic perception, lightweight object detection algorithms based on enhanced You Only Look Once (YOLO) architectures enable real-time detection on embedded robotic platforms^4^. The field has evolved through distinct developmental phases, progressing from traditional handcrafted feature-based methods to modern deep learning approaches that have fundamentally transformed object detection capabilities^5–7^.

The development of deep learning-based object detection has been largely driven by two main technical pathways. Early breakthroughs were dominated by CNNs, which introduced novel detection paradigms. These include two-stage methods such as the Region-based Convolutional Neural Network (R-CNN) series and single-stage detectors like YOLO and Single Shot MultiBox Detector (SSD). CNN architectures fundamentally transformed the object detection landscape by learning hierarchical feature representations directly from data. More recently, Transformer-based architectures have emerged as competitive alternatives, adapting self-attention mechanisms from natural language processing to computer vision tasks^8,9^.

Contemporary deep learning-based detectors can be broadly categorized into two representative frameworks: those built upon CNNs and those designed with Transformer architectures. CNN-based models benefit from inherent inductive biases such as translation equivariance and locality, making them highly effective and efficient for extracting hierarchical visual features. Their success is attributed to sophisticated designs, including Feature Pyramid Networks (FPNs) for multi-scale detection, anchor-based and anchor-free mechanisms for bounding box prediction, and various attention modules for feature enhancement. In comparison, Transformer-based methods leverage self-attention mechanisms to capture global contextual information, demonstrating strong performance in modeling long-range dependencies. These models, such as the pioneering Detection Transformer (DETR)^10^, eliminate the need for many hand-designed components like non-maximum suppression (NMS), thereby offering a more streamlined detection pipeline. The Transformer architecture excels at capturing global context and modeling relationships between distant image regions, making it particularly effective for complex scenes with multiple interacting objects^11,12^.

Multi-modal object detection represents another significant advancement. Integrating complementary information from different sensors and data modalities has proven essential for robust perception in challenging environments. Strategies that fuse visual data, notably RGB, with other inputs including LiDAR, language, or audio have been developed to enhance the functionality and robustness of modern detectors^13^. These approaches enable more comprehensive scene understanding by leveraging the strengths of each modality while mitigating their individual limitations. For instance, LiDAR data provides precise depth information to complement RGB images, while language embeddings offer semantic context that enhances object recognition and enables open-vocabulary detection capabilities.

Despite considerable progress, object detection under real-world conditions still faces multiple challenges. These include the following: handling large scale variations, severe occlusions, and complex backgrounds; maintaining real-time speed on resource-constrained hardware; and achieving seamless multi-modal alignment. Small object detection remains particularly difficult due to limited visual information and low feature resolution. Furthermore, the effective generalization of models to unseen domains and environmental conditions poses significant difficulties. Resolving these issues is crucial for deploying reliable detection systems in practical scenarios.

This review provides a systematic analysis of recent deep learning-based object detection methods, with particular emphasis on CNN and Transformer architectures, along with multi-modal fusion techniques. Unlike previous surveys that often focus on a single paradigm, our work delivers a comprehensive comparison across different technical routes, highlighting their evolutionary trajectories and complementary strengths. We compare the strengths and limitations of each paradigm, summarize key technological advances, identify open challenges, and outline promising research directions. Specialized detection scenarios, including small object detection, oriented object detection, and three-dimensional (3D) object detection, are also examined to provide insights into domain-specific solutions and adaptations. By organizing the rapidly growing literature into a coherent taxonomy, this survey is intended to serve as a valuable reference for researchers and practitioners in the field.

The main contributions of this survey are summarized as follows:

1. A systematic and up-to-date review of evolving paradigms in object detection, with balanced coverage of CNN-based and Transformer-based architectures, as well as emerging multi-modal fusion techniques.
2. A structured comparison and analysis of the strengths and limitations of different detection frameworks, highlighting their evolutionary trajectories and complementary nature.
3. A detailed examination of specialized detection scenarios and domain-specific adaptations, providing insights into practical challenges and solutions.
4. Transparent documentation of both field-wide limitations and review-specific constraints, discussion of persistent challenges, and an outline of promising future research directions.

The remainder of the paper is structured as follows. “Object detection method based on CNN” section reviews object detection methods based on CNNs, while “Object detection method based on transformer and multi-modal fusion” section introduces approaches built upon Transformer architectures and multi-modal fusion techniques. “Performance analysis and benchmark comparison” section presents a performance analysis and benchmark comparison of representative frameworks. “Key technology innovations” section summarizes key technology innovations in the field. “Limitations and challenges” section discusses persistent limitations and challenges, and “Future research directions” section explores promising future research directions. Finally, “Conclusions” section concludes the survey.

## Related works

The field of object detection has undergone rapid development driven by continual architectural innovations and paradigm shifts. This section provides a focused review of SOTA approaches prominent in 2024–2025, organized into three major research directions: CNN-based detectors, Transformer-based frameworks, and multi-modal fusion methods.

### CNN-based object detection

CNN-based detectors remain highly competitive in real-time applications, particularly due to sustained progress in architectural refinement and computational optimization. Recent work has emphasized lightweight designs tailored for edge and embedded deployment. For instance,^14^ presents a computationally efficient network for UAV-based detection that achieves a favorable speed–accuracy trade-off on resource-constrained platforms. Likewise,^15^ proposes a method that effectively handles objects with complex and multiple deformations through adaptive feature extraction modules. For industrial scenarios,^16^ integrates novel attention mechanisms to enhance robustness under challenging environmental conditions. Collectively, these methods exemplify a broader trend toward domain-specific CNN optimization while preserving high computational efficiency.

The YOLO family has also continued to evolve, with its latest variants demonstrating notable gains in both accuracy and robustness across diverse application domains. For underwater scenes,^17^ incorporates large-kernel blocks and multi-branch reparameterization to strengthen feature representation and improve detection performance in visually degraded conditions. In traffic environments,^18^ develops a model dedicated to small object detection by leveraging advanced feature pyramid designs for enhanced multi-scale representation. In the infrared domain,^19^ introduces a strategy for moving small object detection that attains strong performance in aerial vehicle applications. These contributions illustrate the ongoing refinement of single-stage detectors through specialized module design, task-aware architectural customization, and improved training strategies.

### Transformer-based detection frameworks

Transformer-based detectors have reshaped object detection by enabling global context modeling and simplifying end-to-end pipelines. Recent studies have concentrated on improving computational efficiency and enhancing performance in low-data or few-shot regimes.^20^ proposes a hybrid encoder with optimized attention mechanisms that surpasses traditional YOLO-style architectures in real-time detection tasks. For few-shot settings,^21^ presents a language-conditioned detection framework that generalizes to novel categories without retraining, thereby significantly extending its applicability in open-vocabulary scenarios.

Efficiency remains a central research objective for Transformer-based detection.^22^ introduces a softmax-free attention mechanism that reduces computational overhead while maintaining competitive detection accuracy. Complementarily,^23^ employs graph-based token propagation to promote more efficient information transfer within vision Transformers. Together, these works address key computational challenges inherent to standard Transformer architectures while retaining their advantages in global feature modeling and flexible representation learning.

### Multi-modal fusion approaches

Multi-modal object detection has emerged as a pivotal direction for robust perception in complex and dynamic environments. Recent SOTA approaches achieve substantial performance gains by developing more effective cross-modal fusion strategies. For autonomous driving,^24^ implements cascaded cross-attention between RGB and LiDAR modalities in an end-to-end framework, achieving state-of-the-art results on the nuScenes benchmark. In the context of 3D object detection,^25^ leverages hierarchical contrastive learning to strengthen multi-modal feature alignment and improve detection quality.

Beyond autonomous driving, multi-modal fusion has also advanced applications in medical imaging, video analysis, and scene understanding. In medical imaging,^26^ utilizes sequence feature pyramids combined with attention mechanisms to enable precise organ and lesion segmentation across heterogeneous modalities. For audio-visual understanding,^27^ proposes a multimodal pyramid attentional network that delivers superior performance in audio-visual event localization tasks. Most recently,^28^ demonstrates effective integration of visual and linguistic cues to improve detection in text-rich environments. These works collectively underscore the growing importance of cross-modal alignment, complementary feature exploitation, and synergistic fusion strategies in pushing the frontier of object detection across diverse application domains.

**Fig. 1:**
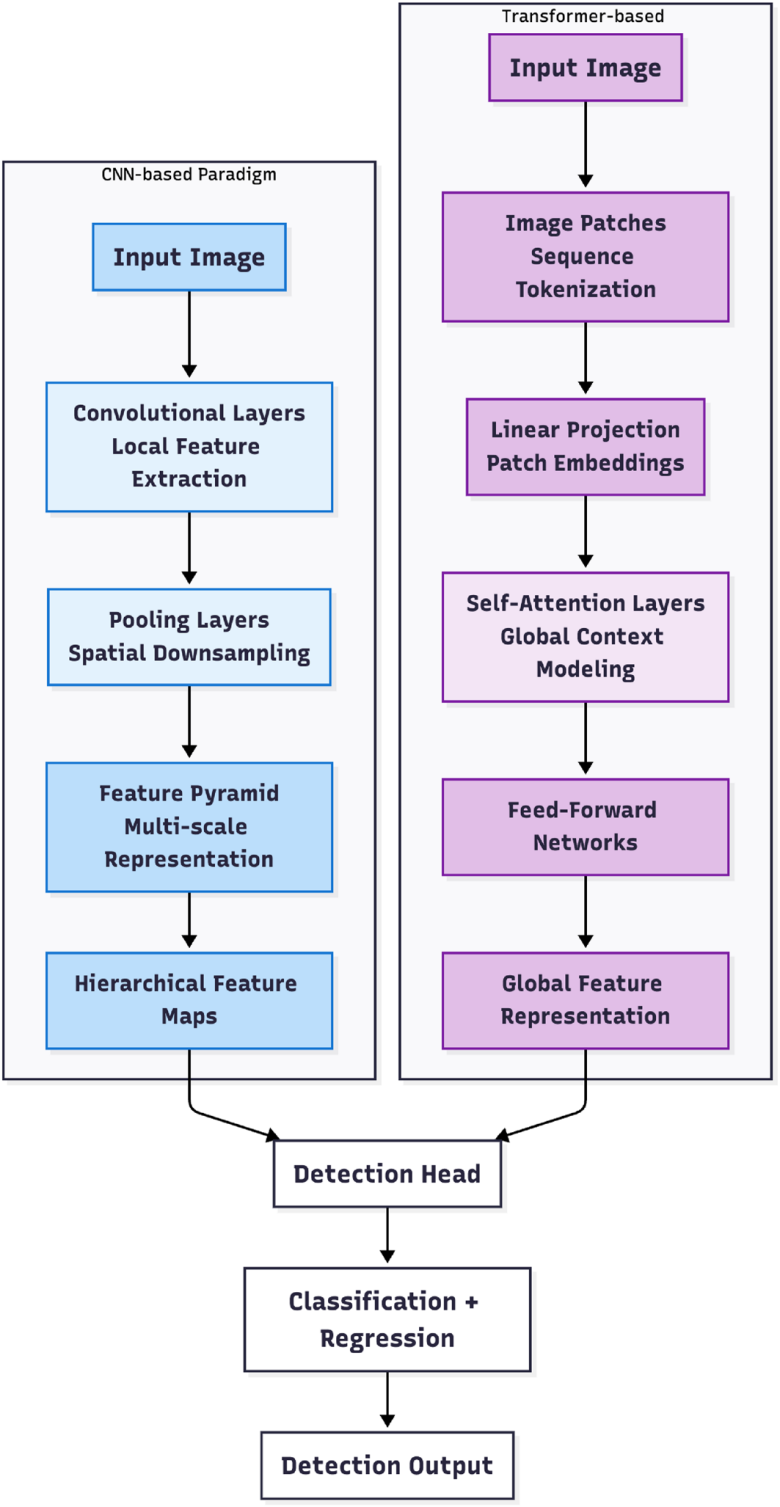
Comparison of CNN-based and Transformer-based detection paradigms. CNN architectures rely on hierarchical convolutional processing with local receptive fields, while Transformer models employ self-attention mechanisms for global context modeling. Both paradigms ultimately feed into detection heads for classification and bounding box regression.

At the conclusion of this literature review, it is instructive to contrast the fundamental architectural principles underlying CNN-based and Transformer-based detectors. As illustrated in Figure 1, CNN architectures employ hierarchical convolutional processing with inherently local receptive fields, while Transformer models utilize self-attention mechanisms to establish global contextual relationships across the entire image. This paradigmatic distinction frames the detailed examination of each approach in the following sections.

## Object detection method based on CNN

### Framework optimization

Current object detection frameworks are primarily categorized into two-stage and one-stage architectures. The fundamental distinction between these paradigms is determined by their region proposal generation mechanisms. In two-stage frameworks, region proposals are generated through a Region Proposal Network (RPN), whereas one-stage models directly predict candidate bounding boxes on feature maps. When an input image is processed by a convolutional neural network (CNN)-based backbone, a comprehensive feature representation is produced. These feature maps are subsequently fused at multiple scales through a neck module, enabling multi-scale feature extraction.

In one-stage models such as YOLOv5, the detection process comprises two principal stages: classification of anchor boxes followed by refinement of bounding box positions using positive samples from multi-scale feature maps. Redundant detections are eliminated through NMS to produce final results. Substantial performance improvements have been demonstrated in recent YOLO-based frameworks through systematic optimizations in backbone architectures, anchor designs, and feature encoding mechanisms. For instance, a lightweight YOLOv5 variant was developed^29^ through integration of C3Ghost and GhostConv modules, effectively reducing computational complexity while improving detection speed. Enhancements to the regional feature encoding module^30^ were achieved through modifications to both FPN and Path Aggregation Network (PANet), thereby strengthening multi-scale feature interactions.

**Fig. 2:**
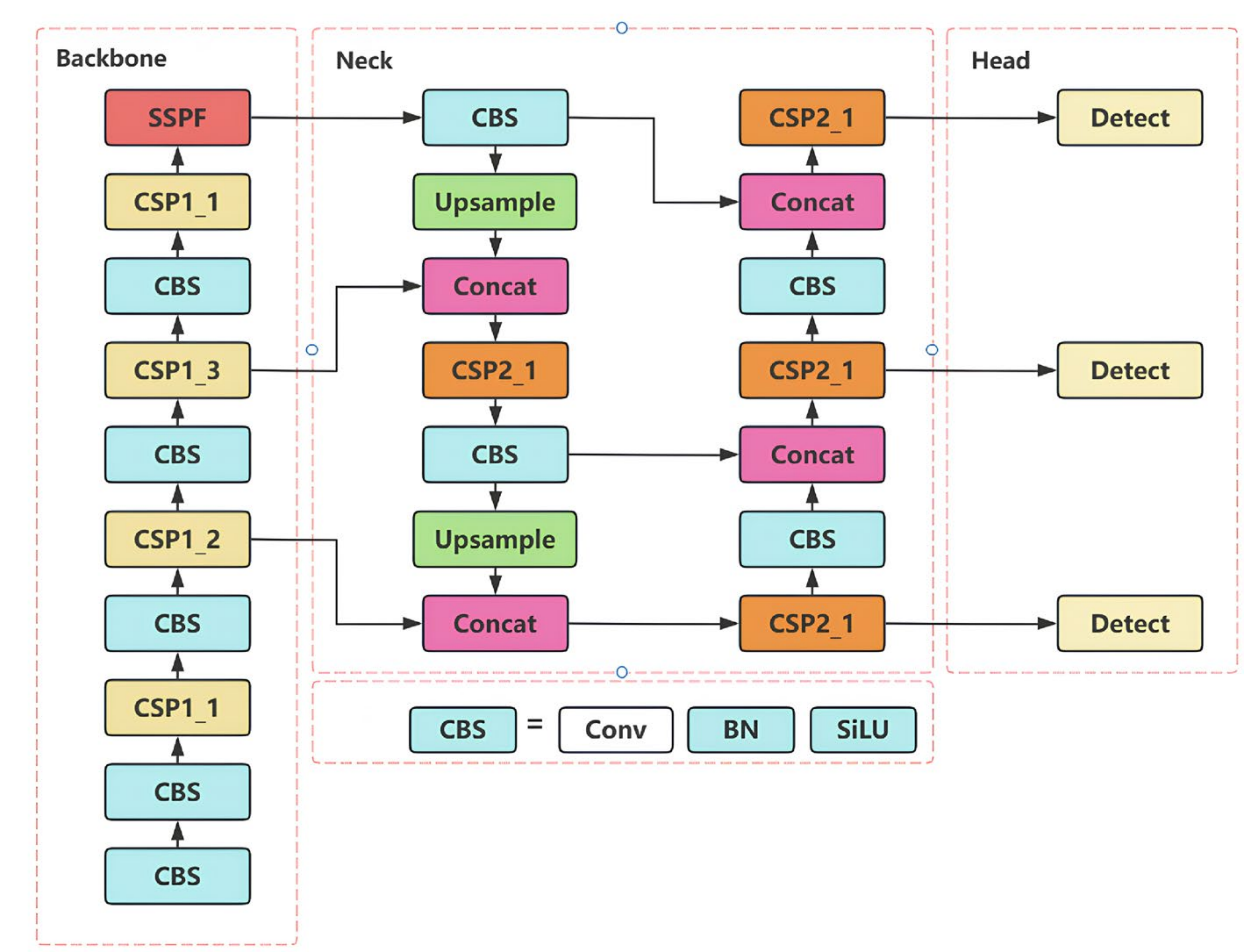
YOLOv5 network architecture.

A coordinate attention mechanism was implemented^31^ to refine spatial localization in feature encoding. Additionally, an innovative inner convolution block was introduced^32^ to augment cross-channel information interactions. To address small object detection challenges, an auxiliary prediction head was specifically designed for processing higher-resolution feature maps in robotics applications. Furthermore, conventional convolution layers in YOLOv5 were replaced with Ghost convolution operations^33^, achieving simultaneous reduction in model parameters and enhancement of robustness. The complete architecture of YOLOv5 is presented in Fig. 2.

Significant improvements in YOLOv8 have been realized through comprehensive architectural refinements to both backbone network and feature extraction modules. Deeper convolutional neural networks and depthwise separable convolutions have been incorporated, enabling superior performance in detecting small and multi-scale targets. Substantial advancements in detection accuracy have been observed compared to YOLOv5, particularly in complex background scenarios. These performance gains are attributed to enhanced capacity for distinguishing overlapping objects. The CSPDarknet network has been optimized to increase inference speed and computational efficiency, while a more efficient training optimization algorithm has been integrated to reduce both training duration and resource consumption. Consequently, YOLOv8 has demonstrated superior performance across multiple metrics including detection accuracy, inference speed, and operational flexibility.

Further backbone optimizations in YOLOv8 have been introduced through the LarK block within UniRepLKNet, expanding the receptive field without increasing model depth. The Modified Penalty Intersection over Union (MPDIoU)^34^ effectively addresses positioning inaccuracies and boundary ambiguities in underwater detection scenarios^35^. Furthermore, integration of Deformable Convolutional Networks version 2 (Deformable ConvNets v2) and coordinate attention mechanisms improves detection of irregularly shaped targets in challenging environments. The replacement of the conventional Darknet-53 backbone with the FasterNet-T0 lightweight variant^36^, substantially reduces computational complexity without sacrificing detection accuracy.

**Fig. 3:**
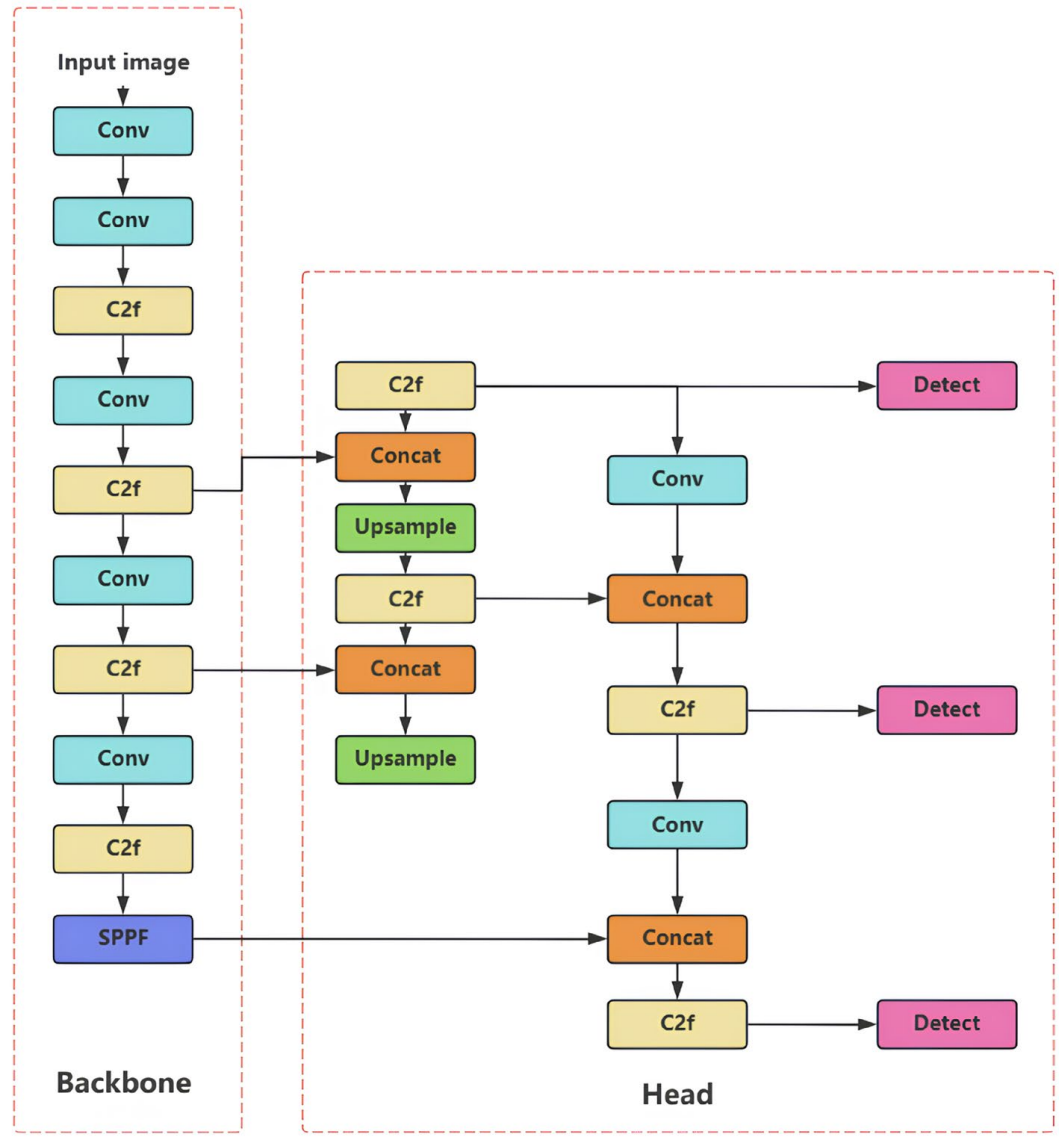
YOLOv8 network architecture.

The Efficient Multi-Scale Attention (EMA) module has been embedded within the C2f-EMA framework to enhance feature extraction through dynamic weight redistribution. The CIoU loss function has been superseded by Powerful-IoU (PIoU)^37^, introducing a penalty term based on bounding box corner discrepancies. An efficient multi-input strategy has been implemented utilizing optical flow images and background-suppressed images as auxiliary inputs. The BiFormer module and lightweight GSConv have been integrated^38^ to prioritize critical target features while maintaining computational efficiency. Additionally, pseudo-multi-channel grayscale image generation has been employed to augment channel information, while network reparameterization techniques have been applied to enhance detection performance without increasing inference latency^39^. The complete YOLOv8 architecture is depicted in Fig. 3.

The two-stage object detection framework is characterized by sequential proposal generation and refinement processes. Initially, candidate regions are generated through a RPN that classifies and regresses bounding boxes. These proposals are subsequently encoded for feature extraction, followed by additional classification and regression procedures. Finally, NMS is applied to eliminate redundant detections. Historically, this framework has been recognized for achieving superior detection accuracy and recall rates compared to single-stage methods, attributed to its multi-step refinement process.

The seminal Faster R-CNN framework^40^ integrated candidate box generation and object detection into a unified end-to-end architecture, significantly enhancing both efficiency and accuracy. However, the RPN has been identified as a computational bottleneck due to excessive region proposal generation. To address this limitation, an RPN filtering algorithm was developed^41^ that combines LiDAR-based and image-based proposals, accelerating sensor fusion while maintaining precision.

Further enhancements include domain adaptation and robustness improvements. Unbiased Faster R-CNN^42^ was proposed to mitigate performance degradation in unseen domains through explicit accounting of data and feature biases. To combat texture interference, Gabor filters were integrated into Faster R-CNN^43^, leveraging frequency analysis capabilities through hybrid training. Architectural refinements have also been investigated, including replacement of the VGG16 backbone with multi-cascade networks^44^ to improve feature extraction for fine-grained details. Additionally, RoIPooling was superseded by RoIAlign to eliminate quantization errors during feature resampling.

### Single-stage object detection framework

In contrast to the two-stage methodology, the single-stage framework performs direct classification and regression of bounding boxes on feature maps, yielding reduced algorithmic complexity and accelerated inference speeds. Although single-stage detectors traditionally exhibited accuracy limitations, recent innovations in multi-scale feature fusion and dynamic anchor mechanisms have substantially narrowed the performance gap between these competing paradigms.

**Fig. 4:**
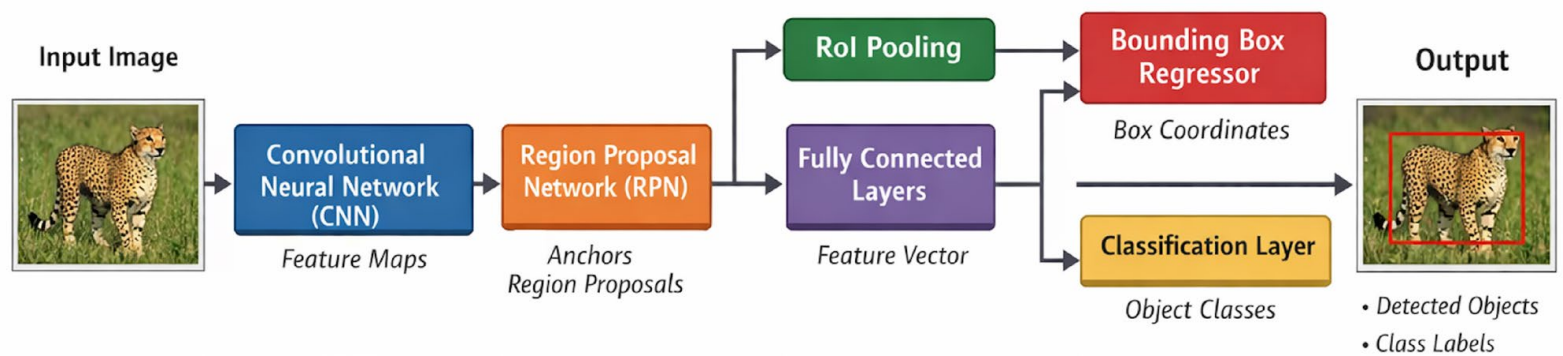
Architecture of Faster R-CNN framework.

The architectural distinction between these approaches is demonstrated in Fig. 4, where Faster R-CNN integrates the RPN and detection network into a unified architecture, contrasting with the streamlined design characteristic of single-stage models such as YOLO. This structural comparison highlights the inherent trade-offs between computational efficiency and detection precision that define the two paradigms.

**Fig. 5:**
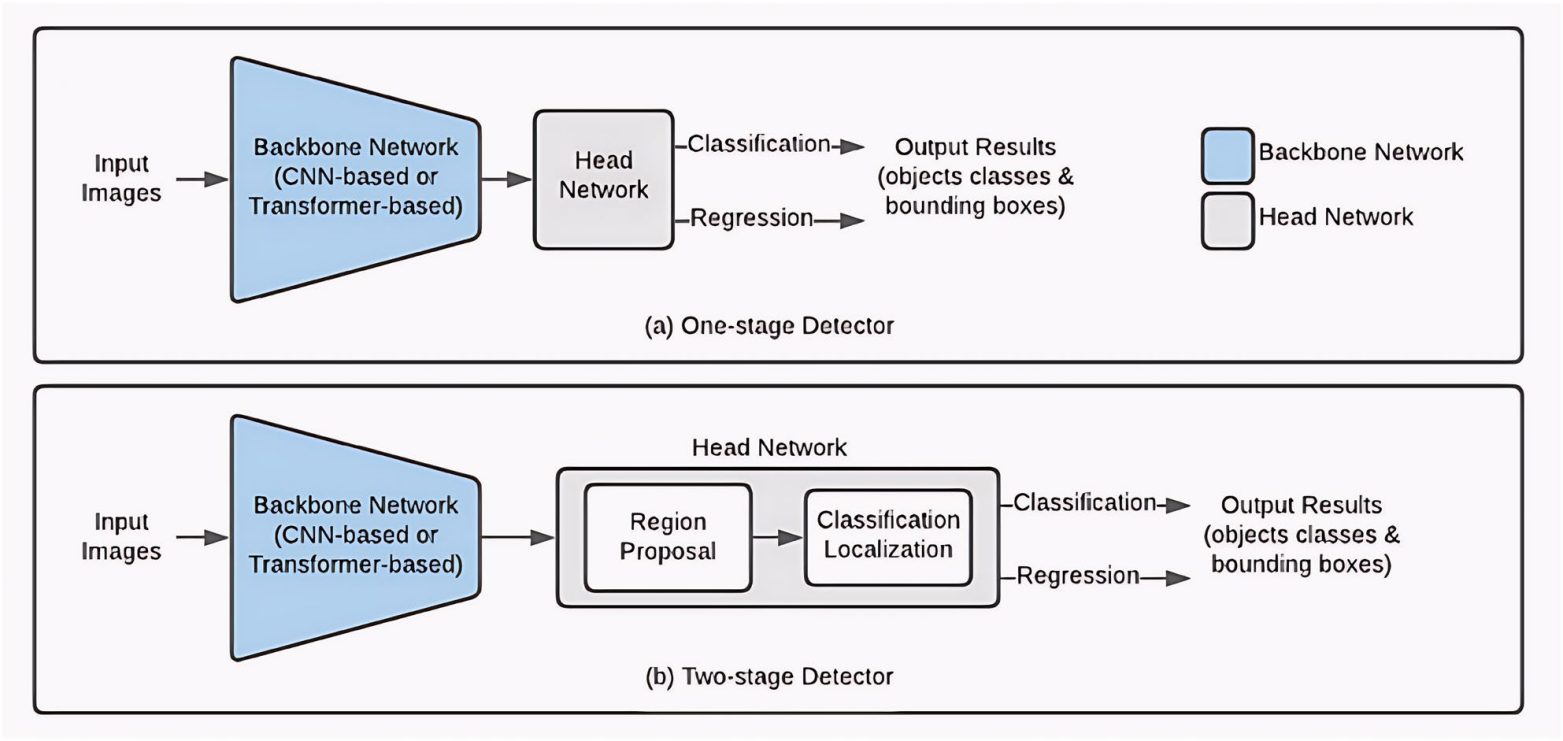
Pipeline comparison between two-stage and single-stage detectors. Two-stage methods (left) employ separate region proposal and refinement steps, while single-stage detectors (right) perform direct classification and regression on feature maps, offering reduced complexity and faster inference.

Although single-stage detectors traditionally exhibited accuracy limitations, recent innovations in multi-scale feature fusion and dynamic anchor mechanisms have substantially narrowed the performance gap between these competing paradigms. The pipeline comparison in Fig. 5 visually articulates the trade-offs between computational efficiency and detection precision that define these two architectural families.

## Object detection method based on transformer and multi-modal fusion

**Fig. 6:**
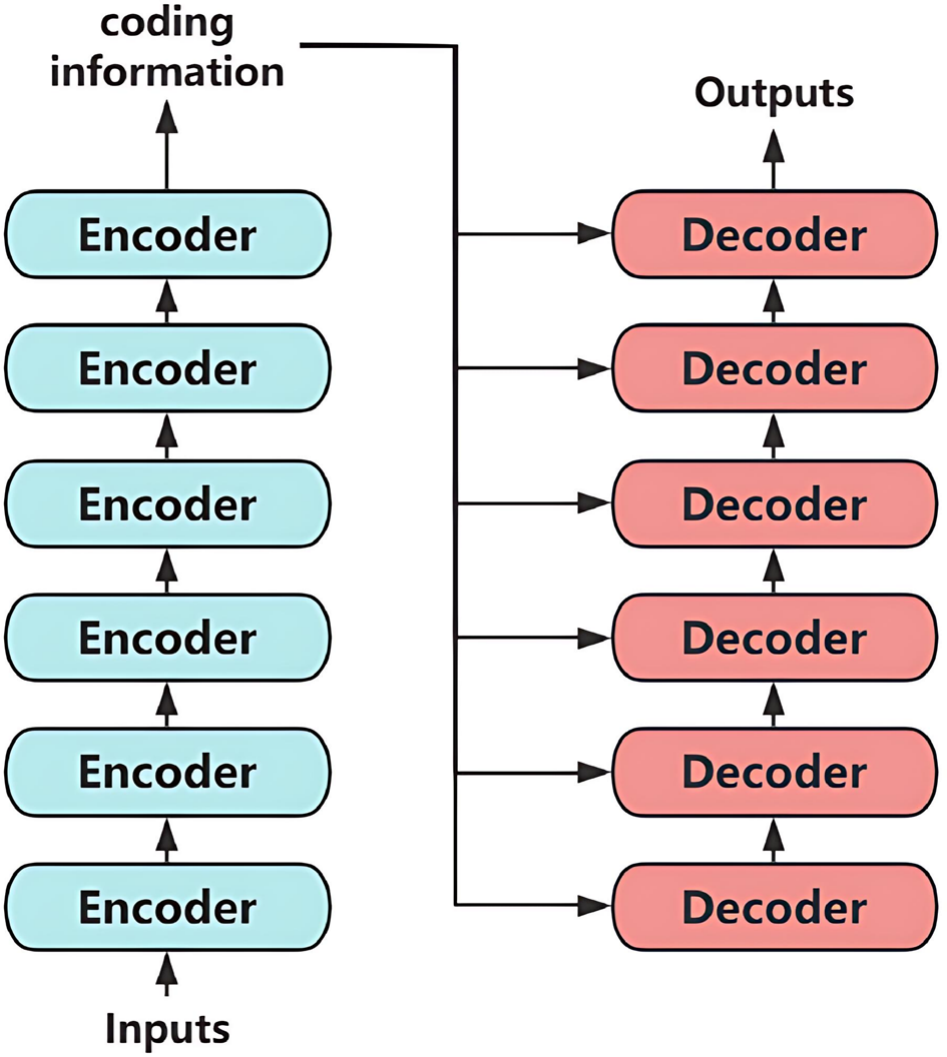
Complete architecture of the Transformer model.

**Fig. 7:**
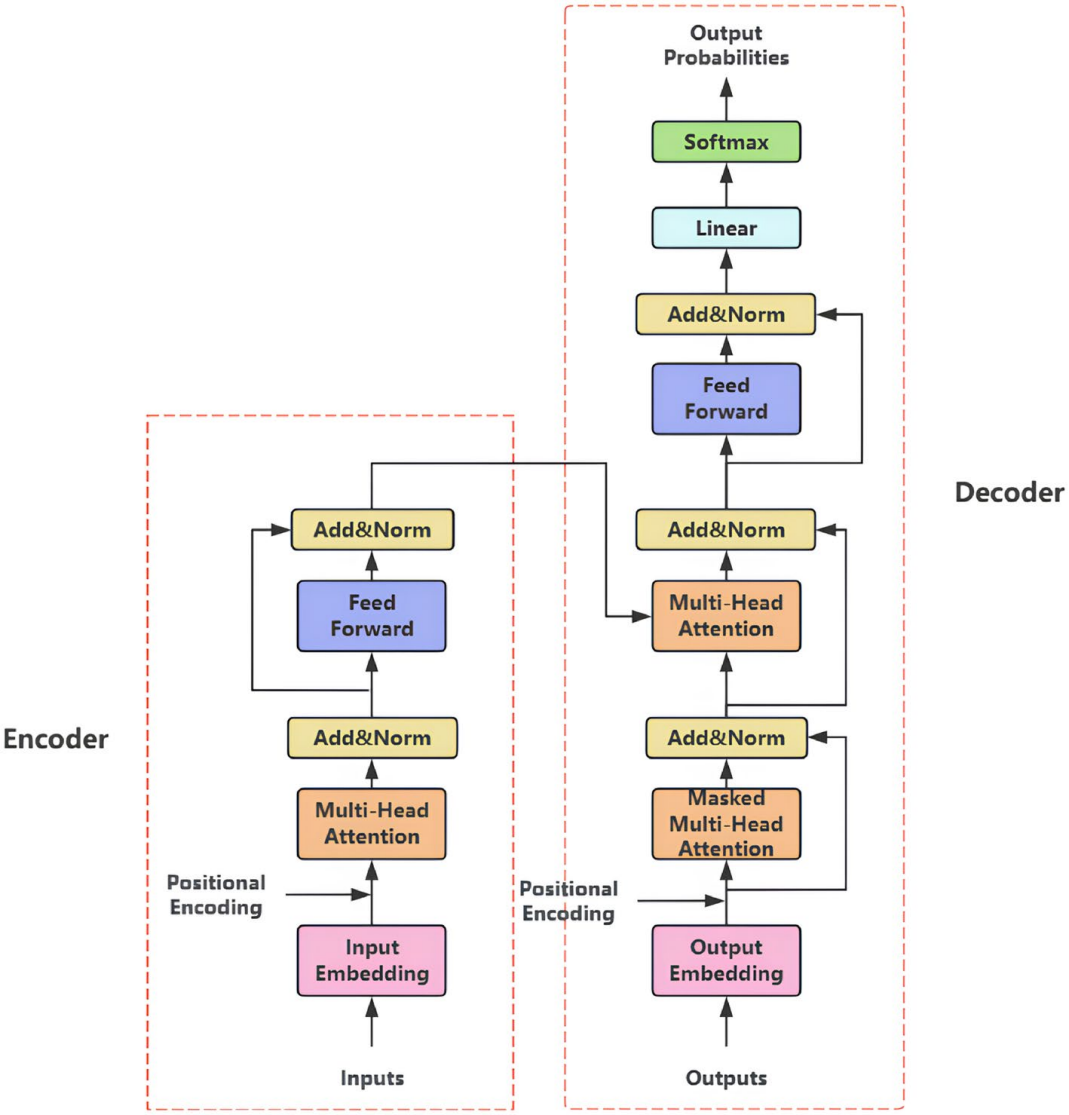
Detailed transformer network architecture.

The Transformer architecture, originally introduced by Vaswani et al.^45^ for sequence processing tasks, has revolutionized numerous domains including natural language processing and computer vision. As depicted in Fig. 6, the fundamental encoder-decoder structure employs multi-head self-attention layers to enable global context modeling through parallel computation. The detailed architectural components are further elaborated in Fig. 7.

**Fig. 8:**
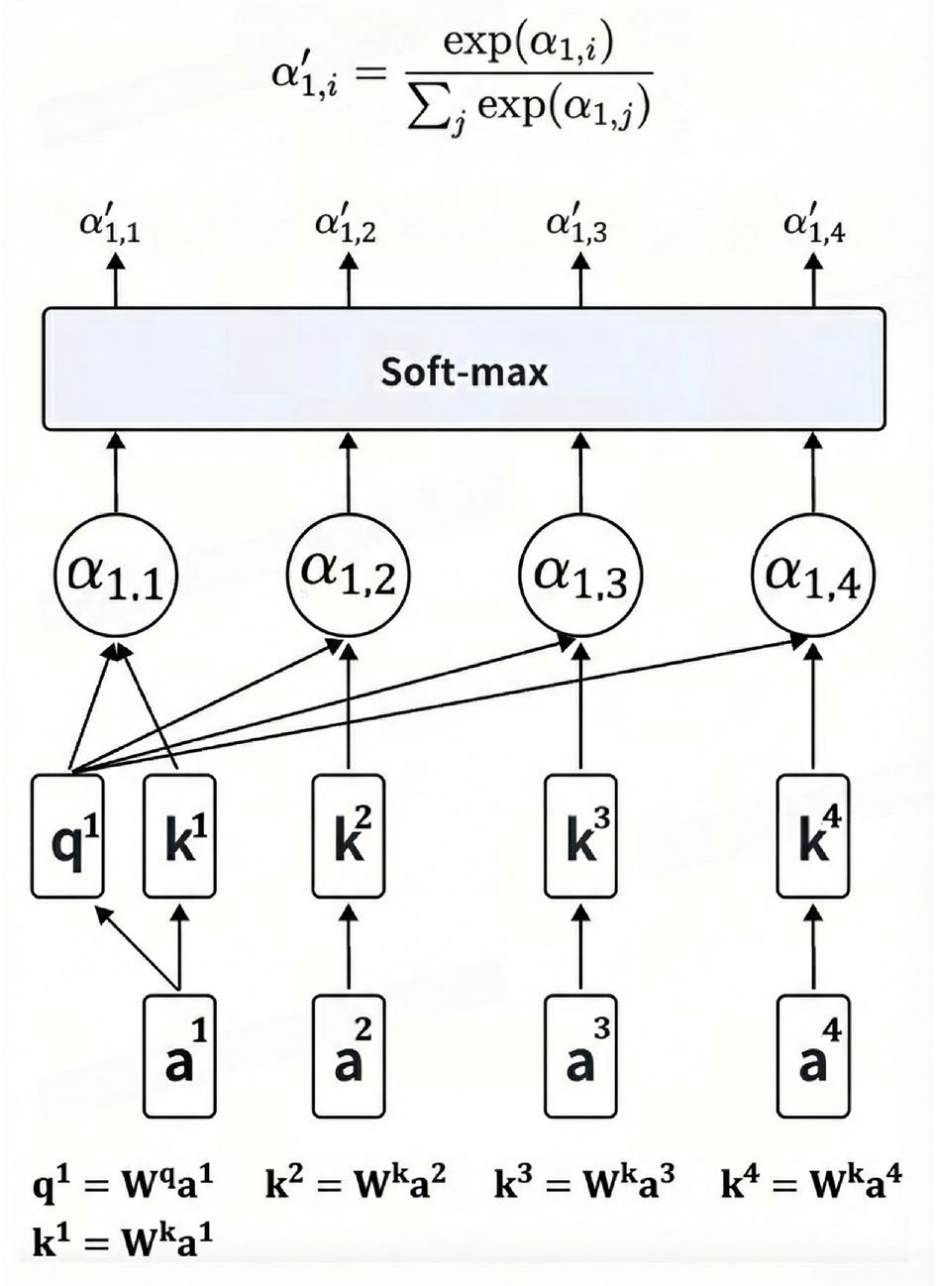
Self-attention mechanism architecture .

Compared to traditional recurrent neural networks (RNNs) and CNNs, the Transformer architecture demonstrates superior efficiency in capturing long-range dependencies within sequential data. This capability originates from the self-attention mechanism, illustrated in Fig. 8, which computes pairwise relationships between all input elements simultaneously. By eliminating the sequential processing constraints inherent in RNNs, this mechanism enables full parallelization during training while maintaining comprehensive awareness of global contextual information.

Recent advancements in Transformer-based attention mechanisms have substantially improved computational efficiency and detection performance. Conventional encoder architectures process all input tokens uniformly, resulting in significant computational overhead. A Softmax-free attention mechanism was proposed by Koohpayegani et al.^46^, reducing computational complexity through $\ell _1$-norm normalization of query-key matrices prior to matrix multiplication. Focus-DETR was introduced by Zheng et al.^47^ to prioritize informative tokens, thereby balancing computational efficiency with model accuracy. Additionally, a graph-based token propagation method was presented by Xu et al.^48^, facilitating information transfer between spatially and semantically connected tokens through adaptive graph edges.

For few-shot learning scenarios, FS-DETR was developed by Chen et al.^49^, eliminating retraining requirements during inference through visual prompt integration. Unlike conventional methods necessitating test-time adaptation, FS-DETR achieves few-shot detection of novel categories by aligning visual and textual embeddings. imTED was designed by Liu et al.^50^, transferring complete pre-trained encoder-decoder structures to detection heads while establishing fully pre-trained feature pathways to enhance generalization capabilities.

To address the slow convergence and data-intensive training requirements of DETR, a hybrid strategy was implemented by Wang et al.^51^ combining feature reconstruction loss, backbone freezing, attention masking, and query shuffling techniques. Transformer attention mechanisms were leveraged by Zhang et al.^52^ to develop a feature translation approach that improves detection performance on unseen categories by $14.7\%$ AP, despite increased computational demands. Finally, DECOLA was presented by Lee et al.^53^ as an open-vocabulary detection framework that adapts its internal mechanisms to user-specified concepts via language embeddings. This framework generates refined pseudo-labels through conditional filtering and achieves state-of-the-art performance on the LVIS benchmark with $42.3\%$ AP.

The self-attention mechanism, illustrated in Fig. 8, dynamically models contextual relationships through query-key-value interactions. The computational process initiates with the projection of input embeddings into three distinct vector spaces: queries representing information requirements of the current position, keys encoding content characteristics across all positions, and values containing actual content representations. The core computation involves calculating pairwise similarity scores between queries and keys through matrix multiplication, followed by softmax normalization to generate attention weights. These weights subsequently modulate the value vectors to produce context-aware representations.

The mathematical formulation of the self-attention mechanism is expressed as:

1 $$\begin{aligned} \text {Attention}(Q,K,V) = \text {softmax}\left( \frac{QK^\top }{\sqrt{d_k}}\right) V \end{aligned}$$

where the query-key correlation matrix $QK^\top \in \mathbb {R}^{n \times n}$ quantifies inter-token dependencies through dot product similarity. The scaling factor $\sqrt{d_k}$ stabilizes gradient computation by constraining the magnitude of attention logits, particularly crucial in high-dimensional spaces where unnormalized dot products may lead to vanishing gradients during backpropagation. This design enables simultaneous processing of all input positions while maintaining awareness of global contextual relationships.

Multi-head attention extends the single-head mechanism through parallel computation across multiple independent attention heads, enabling the model to learn heterogeneous features from distinct representation subspaces. Unlike single-head approaches that process all dependencies through a unified perspective, this architecture captures diverse interaction patterns simultaneously, significantly enhancing contextual modeling capabilities.

The computational process for multi-head attention initiates with linear projections of input queries, keys, and values through head-specific parameter matrices $W_i^Q \in \mathbb {R}^{d_{\text {model}} \times d_k}$, $W_i^K \in \mathbb {R}^{d_{\text {model}} \times d_k}$, and $W_i^V \in \mathbb {R}^{d_{\text {model}} \times d_v}$. For each attention head $i \in \{1,...,h\}$, scaled dot-product attention is computed as:

2 $$\begin{aligned} \text {head}_i = \text {softmax}\left( \frac{(QW_i^Q)(KW_i^K)^\top }{\sqrt{d_k}}\right) VW_i^V, \end{aligned}$$

where $d_k$ denotes the dimension of key vectors. The scaling factor $\sqrt{d_k}$ maintains gradient stability during optimization.

The outputs of all *h* attention heads are concatenated along the feature dimension, followed by a final linear transformation with matrix $W^O \in \mathbb {R}^{hd_v \times d_{\text {model}}}$ to synthesize the integrated representation:

3 $$\begin{aligned} \text {MultiHead}(Q,K,V) = \text {Concat}(\text {head}_1, ..., \text {head}_h)W^O. \end{aligned}$$

This design preserves the input dimension $d_{\text {model}}$ while aggregating information from multiple feature subspaces, allowing concurrent modeling of syntactic, semantic, and positional relationships within the sequence.

The multi-head attention mechanism fundamentally enhances model performance through parallelized computation and diversified feature learning. By enabling simultaneous processing across multiple representation subspaces, distinct attention heads specialize in capturing heterogeneous patterns, including long-range dependencies, local structural relationships, and semantic associations, thereby significantly expanding the model’s representational capacity compared to conventional single-head approaches. This architectural innovation proves particularly effective in handling complex multimodal interactions, where different attention heads can focus on modality-specific features while maintaining cross-modal alignment.

Traditional single-modal approaches exhibit inherent limitations due to their reliance on homogeneous data streams. Vision-centric models frequently demonstrate inadequate textual semantic comprehension and affective interpretation capabilities, while text-based methods often fail to provide sufficient spatial contextualization. These modality-specific constraints manifest through three critical challenges: heightened susceptibility to data noise and quality deterioration, insufficient contextual foundation for decision-making processes, and restricted generalization capacity across diverse application domains. Such deficiencies become particularly evident in cross-modal reasoning tasks where unimodal representations cannot deliver adequate discriminative power, often leading to suboptimal performance in real-world scenarios requiring multimodal understanding.

Multimodal Transformers effectively address these limitations through unified attention-based architectures that process heterogeneous data modalities including images, text, audio, and video within shared embedding spaces. Unlike traditional fusion methods, these models leverage cross-attention mechanisms to establish dynamic inter-modal correlations, enabling synergistic information exchange through learnable attention weights. Recent advancements demonstrate superior performance across various domains: vision-language tasks achieve $15\%$–$22\%$ higher accuracy on VQA benchmarks, audiovisual analysis shows $18\%$ improvement in emotion recognition F1-scores, and medical diagnostics report $12\%$ increased anomaly detection precision compared to conventional approaches. The architecture’s ability to jointly model intra-modal features and inter-modal relationships through attention gates has become foundational for modern multimodal systems.

A multimodal detection framework was pioneered by Zhang et al.^54^, hierarchically integrating intra- and inter-modal features through transformer layers. Their method demonstrates $8.7\%$ mAP improvement on COCO-TextVQA by effectively combining visual and linguistic cues. Building upon this foundation, TransFuser was introduced by Chen et al.^55^, implementing cascaded cross-attention between RGB and LiDAR modalities for autonomous driving applications.

Emerging innovations continue to advance multimodal learning boundaries. The CAT-Det framework^56^ incorporates hierarchical contrastive learning for data augmentation while maintaining $85\%$ computational efficiency relative to baseline models. For temporal interaction analysis, a personality recognition system was developed by Wang et al.^57^ using sliding-window cross-attention, achieving $89.2\%$ F1-score on dyadic conversation datasets. Most notably, a universal fusion transformer was proposed by Liu et al.^58^ that accommodates arbitrary modality combinations through adaptive attention masking and novel contrastive losses, demonstrating $12.9\%$ average performance gains across six multimodal benchmarks.

### Taxonomy of multi-modal fusion strategies

**Fig. 9:**
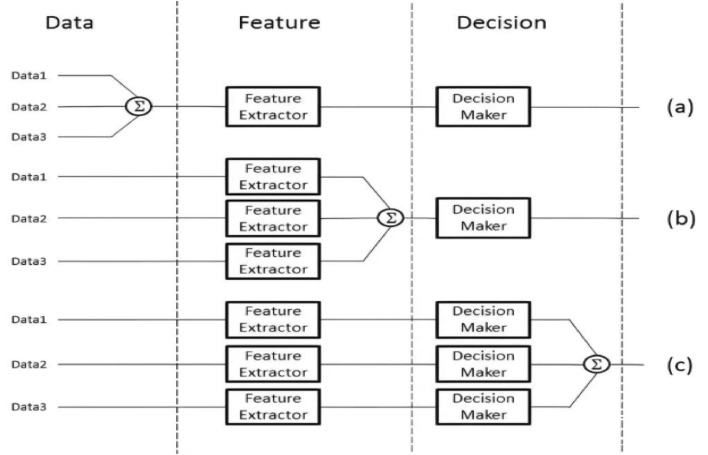
Taxonomy of multi-modal fusion strategies in object detection, illustrating early, middle, and late fusion paradigms with representative models and standard benchmark datasets.

Multi-modal fusion strategies in object detection can be systematically categorized into three paradigms based on the stage at which fusion occurs: **early fusion**, **middle fusion**, and **late fusion**. Each paradigm exhibits distinct architectural characteristics, computational implications, and suitability for different application scenarios. Figure 9 provides a visual taxonomy of these fusion strategies alongside representative models and their associated benchmark datasets.

#### Early fusion

Early fusion integrates raw or minimally processed data from multiple modalities prior to feature extraction. This strategy preserves rich low-level information and enables direct cross-modal alignment at the data level. However, early fusion requires careful synchronization and registration across modalities and exhibits heightened sensitivity to modality-specific noise and artifacts.

Representative approaches include **PointFusion** ^59^, which concatenates RGB images and LiDAR point clouds at the input level for joint 3D object detection, and **AVOD** ^60^, which aggregates multi-modal features from region proposals to enable early cross-modal reasoning. These methods are typically evaluated on large-scale autonomous driving benchmarks such as **KITTI** ^61^, which provides synchronized LiDAR and camera data, and **nuScenes** ^62^, which offers comprehensive multi-modal data including LiDAR, radar, and multiple camera streams.

#### Middle fusion

Middle fusion combines modality-specific features at intermediate network layers following independent feature extraction. This strategy enables adaptive, learned interactions between modalities while allowing each modality to develop specialized representations. Middle fusion has emerged as the dominant paradigm in contemporary multi-modal detectors, balancing flexibility with computational efficiency.

Notable exemplars include **TransFuser** ^63^, which leverages transformer-based cross-attention mechanisms to dynamically align and fuse RGB and LiDAR representations, and **CAT-Det** ^64^, which incorporates contrastive learning to align multi-modal features in a shared embedding space. These approaches are frequently evaluated on **ScanNet** ^65^, a large-scale RGB-D dataset for indoor 3D scene understanding, and other multi-modal benchmarks that support the development of robust fusion mechanisms.

#### Late fusion

Late fusion aggregates detection outputs from independently trained single-modality detectors. This modular approach offers significant robustness to missing modalities and simplifies system deployment, as each modality pathway operates autonomously until the final aggregation stage. However, late fusion inherently sacrifices opportunities for low-level cross-modal interaction and feature synergy.

Representative methods include **CLASTA** ^66^, which combines detection results from LiDAR and camera streams through confidence-based weighting and resolution mechanisms, and multi-view 3D detectors that fuse 3D bounding box proposals from multiple sensor modalities at the output level. These approaches are evaluated on comprehensive autonomous driving benchmarks including **KITTI** and the **Waymo Open Dataset** ^67^, which provide diverse multi-sensor data and challenging real-world scenarios for assessing late fusion robustness.

## Emerging frontiers: unified perception and foundation models

The rapid evolution of object detection has precipitated two transformative trends that are fundamentally redefining the field: the emergence of **unified perception** frameworks and the advent of **large-scale foundation models**. This section analyzes these paradigmatic shifts, their core principles, and implications, while clarifying the unique scope and contribution of this survey within the expanding landscape of detection research.

**Fig. 10:**
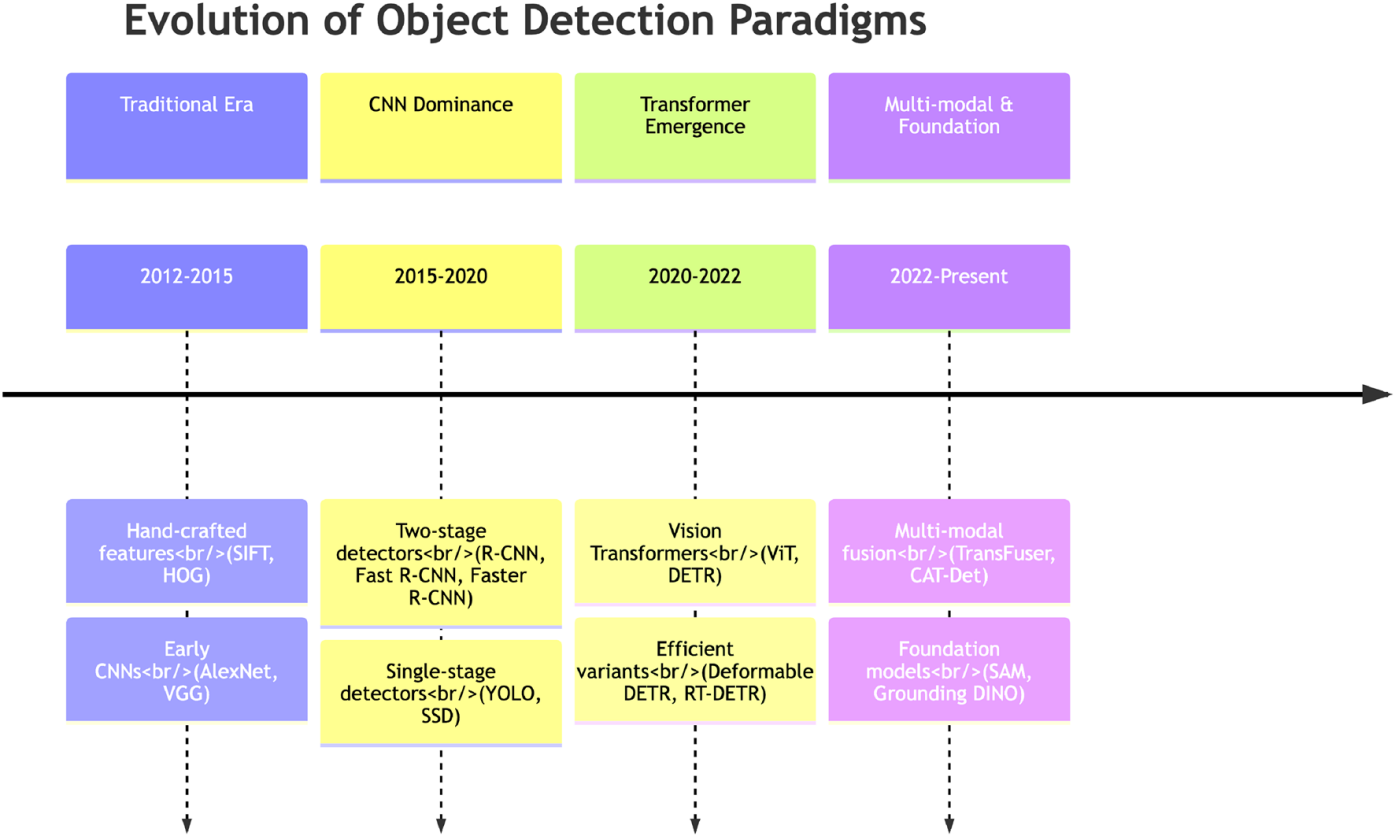
Evolutionary timeline of object detection paradigms, illustrating the transition from traditional methods through CNN-based approaches to Transformer architectures and contemporary foundation models.

As summarized in the evolutionary timeline of Fig. 10, object detection has undergone successive paradigm shifts over the past decade. This historical context is essential for understanding the current frontier developments examined in this section, particularly the emergence of unified perception architectures and foundation models that represent the culmination of preceding technological trajectories.

### From modular detection to unified perception

A fundamental paradigm shift is underway, transitioning from isolated object detection tasks toward unified architectures that jointly address multiple vision tasks, including detection, segmentation, and captioning, within a single end-to-end framework. Enabled by the representational flexibility of Transformer architectures, these models pursue a unified visual understanding across diverse perception objectives.

Representative models such as **UniPercept**^68^ and **UniDet**^69^ exemplify this trend. They employ unified task vocabularies or output embeddings that enable diverse task execution without modifying the core backbone architecture. The primary advantage resides in enhanced knowledge sharing across tasks and a more efficient model ecosystem for deployment. However, these unified models typically exhibit accuracy trade-offs compared to specialized detectors, reflecting a fundamental tension between architectural universality and task-specific peak performance.

### The impact of large-scale foundation models

Since 2023, large-scale foundation models pre-trained on web-scale corpora have fundamentally altered the object detection paradigm. Distinct from traditional detectors trained on fixed-category datasets such as COCO, these models demonstrate remarkable generalization capacity and open-world detection capabilities.

The **Segment Anything Model (SAM)**^70^, while conceived primarily as a segmentation model, carries profound implications for object detection. Its promptable interface enables bounding boxes to serve as prompts for generating high-quality segmentation masks, effectively decoupling localization from recognition. This facilitates a complementary workflow wherein traditional detector outputs are refined by SAM for pixel-accurate segmentation. Critically, SAM’s limitation lies in the absence of semantic awareness, thereby distinguishing *object-agnostic segmentation* from *semantic-aware detection*.

Models such as **Grounding DINO**^71^ and **GLIP**^72^ directly integrate vision-language alignment into the detection framework. By aligning visual features with textual embeddings derived from natural language descriptions, they achieve **open-vocabulary object detection (OVD)**. This capability enables object detection and categorization from arbitrary text queries, substantially expanding applicability beyond predefined category vocabularies. Performance on zero-shot and open-vocabulary benchmarks frequently exceeds that of traditionally trained detectors, signifying a transition toward generalizable and scalable perception systems.

### Positioning and differentiation of this survey

Given these rapid developments, it is essential to clarify the scope and distinctive positioning of this survey.

**Core Focus on Architectural Evolution:** This survey provides systematic analysis of the fundamental **architectural progression** from CNN-based to Transformer-based detection frameworks. Rather than cataloging methods, we examine the underlying design principles, core architectural components, and performance trade-offs that constitute both specialized detectors and the foundational backbones of modern foundation models. Comprehending DETR’s Transformer architecture, for instance, is prerequisite to understanding its successors and contemporary state-of-the-art approaches.

**Foundation Models as Evolutionary Culmination:** We contextualize large-scale foundation models not as an isolated research direction, but as the natural **culmination of architectural and learning paradigm trends** synthesized throughout this survey. These models leverage Transformer-based backbones, multi-modal vision-language fusion mechanisms, and self-supervised pretraining strategies—all central analytical themes in our framework.

**Unique Analytical Contribution:** While existing surveys may focus specifically on foundation model ecosystems, this work offers a distinctive perspective by tracing the **historical and technical lineage** that enabled their emergence. We provide essential conceptual scaffolding for understanding *how* and *why* the field evolved toward models such as SAM and Grounding DINO, through structured comparative analysis of the competing architectural paradigms that shaped this trajectory.

Consequently, this survey serves as an indispensable reference for understanding the underlying mechanisms and evolutionary trajectory of object detection, upon which the contemporary foundation model paradigm is erected.

## Performance analysis and benchmark comparison

### Comparative analysis of object detection frameworks

A comprehensive evaluation of representative object detection frameworks is presented based on established benchmark datasets and performance metrics. The analysis focuses on both conventional CNN-based architectures and emerging Transformer-based approaches, providing insights into their respective strengths and limitations across various application domains.

### Standardized evaluation metrics

Object detection performance is typically assessed using several standardized metrics. The mAP serves as the primary evaluation criterion, with mAP@0.5 measuring performance at 50% IoU threshold and mAP@[0.5:0.95] representing the average performance across IoU thresholds from 0.5 to 0.95 with 0.05 increments. Additional metrics include precision-recall characteristics, F1-score optimization, and computational efficiency measures such as FPS and parameter count.

### Performance on public benchmarks

The comparative performance of major object detection frameworks on the MS COCO dataset^73^ is summarized in Table 1. This analysis reveals distinct performance patterns across different architectural paradigms.

**Table 1:** Performance comparison of representative object detection methods on COCO test-dev benchmark.

Method	mAP@0.5:0.95	mAP@0.5	Params (M)	Speed (FPS)
Faster R-CNN	42.7	62.9	136.7	7
YOLOv5	48.2	66.9	46.5	122
YOLOv8	50.2	68.9	43.7	118
DETR	42.0	62.4	41.3	28
Deformable DETR	46.9	65.7	40.0	19
RT-DETR	53.1	71.3	42.6	108

The performance comparison demonstrates clear trade-offs between detection accuracy and computational efficiency. Single-stage detectors, particularly the YOLO series, achieve superior inference speeds exceeding 100 FPS while maintaining competitive accuracy levels. Transformer-based approaches, including DETR variants, demonstrate progressive improvements in accuracy through architectural refinements, with RT-DETR achieving state-of-the-art mAP@0.5:0.95 of 53.1% while maintaining real-time performance capabilities.

### Domain-specific performance characteristics

Specialized object detection tasks exhibit unique performance patterns across different architectures. Infrared object detection frameworks, evaluated on established benchmarks including NUAA-SIRST, NUDT-SIRST, and IRSTD-1k datasets, demonstrate the effectiveness of Transformer-based approaches in challenging thermal imaging scenarios. As summarized in Table 2, these methods achieve robust performance metrics despite the inherent challenges of infrared imagery.

**Table 2:** Performance comparison on infrared object detection benchmarks (%).

**Metric**	**NUAA-SIRST**	**NUDT-SIRST**	**IRSTD-1k**
IoU	83.21	79.87	81.54
nIoU	88.14	84.36	86.25
$F_{\beta }$	92.45	89.33	90.01

The F-measure, computed as $F_{\beta } = (1 + \beta ^2) \frac{P \cdot R}{\beta ^2 P + R}$ with $\beta =1$, demonstrates balanced precision-recall characteristics across all evaluated infrared datasets. These results highlight the capability of modern detection frameworks to handle domain-specific challenges, including low signal-to-clutter ratio (SCR) scenarios where targets exhibit minimal contrast against background elements.

###  Comparative analysis of SOTA methods

Table 3 presents a systematic comparison of recently published state-of-the-art methods, aggregating reported performance metrics across standard benchmarks. The table summarizes performance on the COCO 2017 test-dev split, utilizing standard metrics including average precision (AP) across different IoU thresholds, computational complexity measured in floating-point operations (FLOPs), and inference speed in FPS. This comparative framework enables multi-faceted evaluation of accuracy-efficiency trade-offs across different architectural paradigms.

**Table 3:** Comprehensive performance comparison of state-of-the-art object detectors on COCO test-dev. (FPS measured on an NVIDIA V100 GPU for 640x640 inputs).

Method	Backbone	mAP@0.5:0.95	mAP@0.5	Params (M)	FLOPs (G)	FPS
*CNN-based Detectors*						
Faster R-CNN	ResNet-101	42.7	62.9	136.7	370	7
YOLOv5l	CSP-Darknet	48.2	66.9	46.5	109	122
YOLOv8m	CSP-Darknet	50.2	68.9	43.7	99	118
*Transformer-based Detectors*						
DETR	ResNet-50	42.0	62.4	41.3	86	28
Deformable DETR	ResNet-50	46.9	65.7	40.0	173	19
RT-DETR-L	HGNetv2	**53.1**	**71.3**	42.6	110	108
*Efficient Detectors for Edge Deployment*						
YOLOv8n	CSP-Darknet	37.3	55.5	3.2	8.7	**285**
YOLO-SM	Custom	41.5	60.1	2.8	6.5	320
LWuAVDet	FasterNet-T0	38.9	57.8	4.1	7.2	295

####  Quantitative performance analysis

The quantitative results in Table 3 reveal distinct performance trade-offs across detector families. Among high-accuracy models, RT-DETR-L achieves 53.1% mAP, representing the highest performance among recently published real-time detectors and reflecting the maturity of Transformer-based architectures. It achieves competitive accuracy with earlier two-stage methods while providing substantial improvements in inference speed. The YOLOv8 series continues to provide excellent balance, with YOLOv8m attaining 50.2% mAP at over 100 FPS. For edge deployment scenarios, recently published lightweight models such as YOLO-SM and LWuAVDet achieve strong performance with 41.5% and 38.9% mAP, respectively, with minimal computational footprint below 10 billion FLOPs, making them suitable for resource-constrained environments.

####  Comparative architectural insights

Beyond quantitative metrics, architectural characteristics reveal distinct behavioral patterns across detector families. Transformer-based models such as RT-DETR and Deformable DETR generally exhibit stronger performance in complex scenes with heavy occlusion and long-range dependencies, benefiting from their global receptive field design. This advantage is reflected in their higher AP scores on the COCO dataset, which contains challenging scenarios. Conversely, highly optimized CNN-based models such as the YOLO series exhibit remarkable robustness and faster inference on standard hardware, making them pragmatic choices for latency-critical applications. According to published benchmark evaluations, modern detectors from both families achieve high-quality bounding box localization, with RT-DETR and YOLOv8 demonstrating particularly low false-positive rates in cluttered scenes.

### Architectural efficiency analysis

Computational efficiency represents a critical consideration in practical object detection systems. The parameter efficiency and inference speed analysis reveals that recent architectural innovations have substantially improved the performance-to-complexity ratio. Lightweight variants of established frameworks, such as YOLOv8n and EfficientDet, achieve compelling performance while maintaining minimal computational footprints suitable for edge deployment.

The evolution of loss functions has also contributed significantly to detection performance improvements. Modern localization losses, including CIoU, EIoU, and MPDIoU, address various aspects of bounding box regression accuracy. These advancements have proven particularly beneficial for challenging detection scenarios involving occluded objects, extreme aspect ratios, and small target sizes.

### Future performance trends

Emerging trends in object detection performance optimization focus on several key directions. Neural architecture search (NAS) techniques are increasingly employed to discover optimal model configurations tailored to specific deployment constraints. Knowledge distillation methods enable the transfer of detection capabilities from large teacher models to efficient student networks without significant performance degradation. Additionally, dynamic inference mechanisms that adapt computational expenditure based on input complexity offer promising pathways for balancing accuracy and efficiency in real-world applications.

The integration of multi-modal information continues to demonstrate substantial performance gains, particularly in challenging environmental conditions. Fusion of complementary data sources, including RGB with thermal, LiDAR, or textual information, enables more robust detection across diverse operational scenarios. These advancements collectively contribute to the ongoing progression of object detection capabilities toward human-level performance across an expanding range of application domains.

## Key technology innovations

The landscape of object detection has been fundamentally reshaped by several pivotal technological advancements that have progressively addressed core challenges in the field. These innovations span architectural paradigms, training methodologies, and multi-modal integration strategies, collectively driving the remarkable progress observed in recent years.

The integration of Transformer architectures represents one of the most transformative developments, with DETR establishing a novel end-to-end paradigm that eliminates the requirement for hand-designed components such as non-maximum suppression. This approach leverages global self-attention mechanisms to capture long-range dependencies across entire images, fundamentally differing from the local receptive fields characteristic of traditional CNNs. Subsequent innovations including RT-DETR and Deformable DETR have addressed computational complexity concerns through hybrid encoders and sparse attention mechanisms, respectively, enabling practical deployment while preserving detection accuracy^74^.

Concurrently, single-stage detectors have undergone substantial refinement through architectural enhancements and training optimization techniques. The evolution of YOLO series architectures has demonstrated consistent improvements in both accuracy and efficiency, with YOLOv5 and YOLOv8 incorporating advanced feature pyramid networks, adaptive anchor mechanisms, and distillation learning approaches. These developments have proven particularly valuable for real-time applications, as evidenced by their successful deployment in multi-UAV collaborative systems requiring efficient feature extraction and trajectory estimation^75^.

Two-stage detection frameworks have similarly benefited from significant methodological advances. Modern implementations of Faster R-CNN variants have incorporated sophisticated feature fusion techniques, including bilinear interpolation and attention modules, to enhance proposal quality in challenging scenarios involving occlusion and complex backgrounds. The introduction of specialized loss functions such as $\alpha ^{*}$-WIoU v3 and architectural components like the BiFormer module have further refined bounding box prediction accuracy, while adaptive attention fusion mechanisms have enabled effective integration of 2D and 3D parsing results^76,77^.

Multimodal detection approaches have emerged as a particularly promising direction, leveraging Transformer-based architectures to align and integrate heterogeneous data streams. The fusion of visual embeddings with linguistic representations has enabled open-vocabulary detection capabilities, substantially expanding the applicability of detection systems to novel categories beyond training distributions. Contrastive learning techniques have demonstrated notable success in establishing shared embedding spaces that facilitate effective cross-modal alignment, thereby enhancing contextual understanding and generalization performance^78^.

The synergistic integration of these technological innovations has progressively narrowed the performance gap between computational efficiency and detection accuracy, while simultaneously expanding the operational envelopes of object detection systems across diverse application domains. Nevertheless, as discussed in the subsequent section, significant challenges persist that continue to motivate ongoing research and development efforts.

## Limitations and challenges

Despite substantial advancements in object detection methodologies, several notable limitations persist that constrain practical deployment in real-world scenarios. These challenges span multiple dimensions: scale variation, environmental complexity, data distribution characteristics, and computational constraints. Scale variation remains a significant challenge, particularly for objects exhibiting extreme size differences within individual scenes. While feature pyramid networks have improved multi-scale processing, performance degradation occurs at dimensional extremes. Small objects suffer from limited spatial resolution and reduced feature information during extraction, while large objects frequently exhibit boundary ambiguity and internal pattern distortion. This proves particularly problematic in applications such as aerial surveillance and autonomous driving, where apparent object sizes vary substantially.

Real-world environments present intricate spatial relationships with frequent partial occlusions that complicate detection. Current detection systems demonstrate limited capability in reasoning about occluded regions and distinguishing targets from semantically similar backgrounds, particularly in cluttered urban scenes. Although attention mechanisms and contextual modeling have provided incremental improvements, a notable gap persists between machine and human visual reasoning in these scenarios. Long-tail distributions characteristic of real-world datasets create significant challenges for learning discriminative features across all categories. Dominant classes disproportionately influence model parameters, while rare classes remain underrepresented. Fine-grained detection tasks are further complicated by visual similarity between semantically distinct categories, where subtle distinguishing features must be reliably identified.

The tension between accuracy optimization and operational efficiency becomes acute in resource-constrained environments. Edge computing applications face strict requirements on latency, memory, and energy consumption. Model compression techniques (including pruning, quantization, and knowledge distillation) offer potential solutions; however, they frequently incur robustness penalties, particularly for challenging detection scenarios involving scale variation, occlusion, or unusual viewing conditions.

Beyond these field-wide limitations, this survey has specific constraints that merit acknowledgment. The scope of literature coverage is necessarily bounded by selection criteria and data availability, potentially affecting generalizability. Performance comparisons rely primarily on metrics reported in original papers rather than unified experimental evaluation under consistent conditions. Multi-modal fusion strategies are discussed conceptually rather than experimentally, with practical deployment challenges remaining inferred rather than empirically validated. Discussion of future research directions is necessarily high-level, without detailed technical implementations or quantitative predictions.

These interconnected challenges collectively underscore the need for holistic approaches addressing multiple aspects of detection robustness and efficiency. By transparently acknowledging both general field limitations and survey-specific constraints, we provide a realistic assessment of the current state-of-the-art. Progress will likely require coordinated advances across architectural design, training paradigms, and domain adaptation strategies, potentially drawing inspiration from biological vision systems while leveraging emerging computational frameworks.

## Future research directions

Building upon current technological advancements and addressing the identified limitations, several promising research trajectories merit focused exploration. These directions span architectural innovations, training paradigm evolution, and deployment optimization strategies, collectively aiming to advance object detection capabilities for real-world applications.

### Architecture design and optimization

The development of efficient network architectures remains crucial for enhancing object detection performance. Future research should focus on designing adaptive backbone networks that dynamically adjust computational pathways based on input characteristics^79,80^. NAS methodologies, particularly those incorporating hardware-aware constraints, present promising approaches for discovering optimal network configurations^81^. The continued refinement of attention mechanisms, especially those enabling spatially adaptive receptive fields, could substantially improve handling of scale variations and occluded objects^82^.

For detection head design, research into unified frameworks that bridge anchor-based and anchor-free paradigms offers significant potential^83^. Adaptive anchor learning strategies, where detectors autonomously select appropriate reference boxes during training, may overcome limitations of manual parameter tuning. Furthermore, investigation of keypoint-based detection approaches could provide alternative solutions that bypass the need for region proposal operations.

### Advanced learning paradigms

Future research should prioritize the development of sophisticated training methodologies that address fundamental challenges in object detection. Meta-learning frameworks enabling rapid adaptation to novel categories and domains warrant deeper investigation, particularly for open-world recognition scenarios^84^. Self-supervised and weakly-supervised learning techniques present promising avenues for reducing annotation dependencies while maintaining detection accuracy^85^.

The integration of causal learning frameworks may yield detectors with improved out-of-distribution performance and enhanced interpretability^86^. Curriculum learning strategies that progressively expose models to increasingly complex scenarios could substantially improve generalization capabilities. Additionally, research into multi-task learning architectures that jointly optimize detection with related vision tasks may lead to more computationally efficient and semantically coherent systems.

### Multi-modal and contextual integration

The effective fusion of heterogeneous data sources represents a critical research direction. Future work should investigate hierarchical fusion frameworks that integrate features across spatial, temporal, and semantic dimensions^87,88^. The development of context-aware detection systems capable of leveraging scene semantics and relational reasoning will be essential for robust performance in complex environments^89^.

Research into cross-modal alignment techniques, particularly for vision-language integration, could substantially expand detection capabilities to novel categories and domains^90^. The exploration of neuromorphic fusion approaches inspired by biological visual systems may offer novel solutions for integrating multi-sensor inputs while maintaining computational efficiency^91^.

### Deployment-oriented optimization

As object detection systems transition to real-world applications, deployment-focused optimization becomes increasingly important. The development of adaptive inference mechanisms that dynamically adjust computational expenditure based on input complexity presents a crucial research direction^92^. Hardware-software co-design approaches optimized for specific computing platforms, including emerging edge AI processors, warrant extensive investigation^93^.

Substantial research opportunities exist in developing comprehensive evaluation frameworks that assess not only detection accuracy but also robustness, fairness, and energy efficiency^94^. Model compression techniques that maintain detection performance while reducing computational requirements remain an active research frontier, particularly for resource-constrained applications.

## Conclusion

This survey has systematically examined the evolution of object detection from CNN-based to Transformer-based architectures and multi-modal fusion strategies. The field has achieved steady progress in addressing scale variation, occlusion handling, and computational efficiency, enabling more balanced trade-offs between accuracy, speed, and robustness. These developments have broadened applicability across autonomous systems, medical imaging, and related domains.

However, substantial challenges persist in achieving human-level visual understanding, particularly under extreme scale variations, complex occlusions, and distribution shifts. Computational constraints, limited domain generalization, and data bias remain significant barriers to practical deployment.

Future progress will require interdisciplinary collaboration among computer vision, neuroscience, and hardware design. Biologically inspired architectures and resource-efficient adaptive learning frameworks show promise in advancing object detection toward greater robustness and real-world applicability.

## Data Availability

This review is based on publicly available benchmark datasets. The primary dataset analyzed is the MS COCO (Common Objects in Context) dataset, which is available at http://cocodataset.org/. All performance comparisons and findings discussed in the manuscript are derived from models trained and/or evaluated on this public dataset.
